# High study participation but diverging adherence levels: qualitatively unpacking PrEP use among adolescent girls and young women over two years in Eastern Cape, South Africa

**DOI:** 10.1007/s10865-023-00462-2

**Published:** 2023-12-11

**Authors:** Emily Krogstad Mudzingwa, Lindsey de Vos, Millicent Atujuna, Lauren Fynn, Matinatsa Mugore, Selly Mabandla, Sybil Hosek, Connie Celum, Linda-Gail Bekker, Joseph Daniels, Andrew Medina-Marino

**Affiliations:** 1 The Desmond Tutu HIV Centre, University of Cape Town, Desmond Tutu Health Foundation, 3 Woodlands Road, Woodstock, Cape Town, 7915 South Africa; 2 Institute for Collaboration on Health, Intervention, and Policy, University of Connecticut, Storrs, CT USA; 3 Research Unit, Foundation for Professional Development, Buffalo City Metro, Eastern Cape Province South Africa; 4 Mailman School of Public Health, Columbia University, New York, NY USA; 5 HIV/AIDS, STIs and TB Directorate, Buffalo City Health District, Eastern Cape Provincial Department of Health, Bisho, South Africa; 6 Department of Psychiatry, Stroger Hospital of Cook County, Chicago, IL USA; 7 Division of Infectious Diseases, Stroger Hospital of Cook County, Chicago, IL USA; 8 Department of Global Health, University of Washington, Seattle, WA USA; 9 Department of Epidemiology, University of Washington, Seattle, WA USA; 10 Edson College of Nursing and Health Innovation, Arizona State University, Phoenix, AZ USA; 11 Department of Psychiatry, Perelman School of Medicine, University of Pennsylvania, Philadelphia, PA USA

**Keywords:** Pre-exposure prophylaxis, South Africa, Adherence, HIV prevention, Adolescent girls and young women

## Abstract

In Southern and Eastern Africa, initiation of daily oral pre-exposure prophylaxis (PrEP) for HIV prevention has been high among adolescent girls and young women (AGYW) offered PrEP. However, persistence and prevention-effective use of PrEP among this critical group continues to be a challenge. We conducted a qualitative sub-study of AGYW from the Community PrEP Study in Eastern Cape Province, South Africa who had high rates of pick up for monthly PrEP refills over two years, but differing levels of PrEP adherence based on tenofovir-diphosphate (TFV-DP) measurements in dried blood spots (DBS). Contrasting 22 AGYW with low versus high levels of TFV-DP in DBS, we qualitatively explored factors which influenced PrEP persistence vs. non-persistence, unique patterns of PrEP use (e.g., discarding or stockpiling), and participant recommendations for improving AGYW prevention-effective use of PrEP in the future. Results showed that PrEP misconceptions and mistrust among participants’ social networks negatively influenced adherence. In comparison, supportive families and/or partners and personal trust that PrEP works positively influenced adherence. Those with low adherence described being motivated to come to the site for other study benefits (e.g., reimbursement money, snacks, sanitary pads) and discarding PrEP to avoid stigma associated with being seen with pills. Future PrEP implementation strategies should focus on involving families and partners in PrEP support for AGYW and minimizing PrEP stigma at a community level.

*Trial registration* NCT03977181. Retrospectively registered on June 6, 2019.

**Supplementary Information:**

The online version contains supplementary material available at 10.1007/s10865-023-00462-2.

## Introduction

Adolescent girls and young women (AGYW) in sub-Saharan Africa remain at high risk for acquiring HIV, accounting for ~ 25% of new infections globally (Celum et al., 2021). Adherence to daily oral pre-exposure prophylaxis (PrEP) containing tenofovir for HIV prevention has continued to be a challenge for many of these AGYW. Although four efficacy trials of oral PrEP demonstrated high efficacy among men who have sex with men, heterosexual serodifferent couples in East Africa, and injection drug users (Baeten et al., 2012; Choopanya et al., 2013; Grant et al., 2010; Thigpen et al., 2012), two efficacy trials of PrEP among African women had flat results, largely attributed to poor adherence (Damme et al., 2012; Marrazzo et al., 2015). Subsequent studies of PrEP implementation among African AGYW have shown high levels of PrEP initiation but rapidly declining adherence within the first six to twelve months (Celum et al., 2020; Celum et al., 2021; Gill et al., 2020; Rousseau et al., 2021). Qualitative research from these studies have shown that reasons for poor adherence have included lack of intrinsic motivation, unsupportive family members and/or partners, challenges developing a routine for PrEP use, and experiences with stigma and community misconceptions (Rousseau et al., 2021; O’Rourke et al., 2021; Giovenco et al., 2021; Stoner et al., 2021; Velloza et al., 2020).

Previous PrEP studies have also reported a large discrepancy between self-reported PrEP adherence (high) and measured drug adherence in blood (low) (Corneli et al., 2014; Marrazzo et al., 2015). Social desirability bias and a desire among study participants to continue receiving other study benefits have been suggested as contributors to this discrepancy (Corneli et al., 2015; Straten et al., 2014). While multiple interventions have been developed to improve PrEP adherence among AGYW, including cash incentives (Celum et al., 2020), drug level feedback (Celum et al., 2021), and peer-based empowerment clubs (Delaney-Moretlwe et al., 2018), few have made a difference in adherence. Consequently, a deeper understanding of the barriers and facilitators to AGYW PrEP persistence or discontinuation, and how study participation uniquely impacts PrEP use is needed. Carefully engaging with and integrating the voices of different AGYW in both rural and urban settings in developing future interventions is essential to improve their prevention-effective use of PrEP.

We describe experiences of a qualitative subset of AGYW participants from the Community PrEP Study (CPS) (Medina-Marino et al., 2021a) who exhibited high rates of return for monthly PrEP refills but had divergent levels of measurable PrEP medication (tenofovir-diphosphate, or TFV-DP) in their blood throughout the two-year study. We explored factors that may have influenced their diverging persistent PrEP use, unique patterns of PrEP use in the study (e.g., stockpiling, discarding, or sharing PrEP), and considered participants’ recommendations for improving PrEP rollout and adherence support for other AGYW in the future. This research addresses two important gaps. First, previous studies have examined shorter-term PrEP adherence among African AGYW from 3 to 12 months (Celum et al., 2021; Gill et al., 2020; Stoner et al., 2021). Here we explore experiences with longer-term PrEP use over two years, stratified by high vs. low adherence. Second, there have been limited reports on what happens to unused PrEP, including selling, sharing, and stockpiling of PrEP (Corneli et al., 2015). Ultimately, we aimed to better understand what factors impact prevention-effective use of PrEP among this population, which may help inform future implementation strategies for both daily oral PrEP and long-acting PrEP.

## Methods

### Study design and setting

The Community PrEP Study (CPS) was a mixed methods study conducted among 603 AGYW aged 16–25 years in Eastern Cape Province, South Africa from 2018 to 2021 to evaluate the impact of community-based platforms for increasing access and adherence to daily oral PrEP for HIV prevention (Medina-Marino et al., 2021a, 2021b, Mudzingwa et al., 2022a, Mudimu et al., 2022b). AGYW were recruited from two communities (one peri-urban, one rural) in the Buffalo City Metro (BCM) Health District. In 2018, Eastern Cape had an estimated HIV prevalence of 12.1% and an incidence of 1.91% among AGYW 16–25 years (Johnson & Dorrington, 2022; Johnson et al., 2017). As of 2017, 15.6% of youth aged 15–24 in Eastern Cape Province reported early sexual debut (before age 15), and 14.4% of children aged 18 and younger had experienced orphanhood (Simbayi et al., 2017). Most residents speak isiXhosa, and approximately 25% of the population in BCM resides in informal housing (Council, 2017).

Details of the CPS protocol and baseline results have been previously published (Medina-Marino et al., 2021a, 2021b). Briefly, AGYW were recruited from community-based HIV testing service (HTS) platforms, including: (1) home-based HTS, and (2) mobile pop-up sites. Upon receiving negative HIV test results, AGYW were referred to study-established community-based PrEP initiation services. AGYW who met inclusion criteria and consented to be part of the study were randomly assigned to one of three study arms (PrEP medication pick-up only; one-on-one adherence counselling; and group adherence counselling), provided same-day PrEP and invited to return for monthly PrEP refills for 24 months. All participants completed a social-demographic and sexual risk behavior survey at study baseline using an ACASI platform and additional behavioral questionnaires at months 3, 12, and 24. PrEP adherence was measured at months 3, 6, 12, 18, and 24 via collection of dried blood spots (DBS) to detect intracellular tenofovir diphosphate (TFV-DP) levels; TFV-DP levels provide an estimate of cumulative dosing over the past four to six weeks (Anderson et al., 2018). Participants were given refreshments, sanitary pads, and offered HIV and pregnancy testing at monthly PrEP refill visits. Additionally, they received 30 ZAR (~ $2 USD) for each visit where a DBS sample was collected and 100 ZAR (~ $7 USD) for each interview. Other health services such as STI testing and Hepatitis B vaccination were provided at pre-specified study visits (Medina-Marino et al., 2021a). This study is reported in accordance with the consolidated criteria for reporting qualitative research (COREQ) guidelines (Supplemental Table 1) (Tong et al., 2007).

### Data collection for qualitative subset

A total of 89 participants were purposively recruited to complete qualitative interviews to further explore their experiences initiating and adhering to PrEP throughout the study. This manuscript examines a subset of (n = 22) these participants who all had high rates of return for monthly PrEP refills, and attended at least three of five study visits in which DBS samples were collected. We then stratified these 22 study participants into two groups: (1) those (n = 7) with high levels of TDF-DP in DBS (≥ 700 fmol/punch) averaged across all their DBS measurements during the study (“High TFV-DP”), and (2) those (n = 15) with low to moderate levels of TDF-DP in DBS (< 700 fmol/punch) averaged across all their DBS measurements during the study (“Low TFV-DP”). This threshold (≥ 700 fmol/punch) correspond to an average of four doses per week in males in the USA (Anderson et al., 2018), the level associated with 100% protection among men who have sex with men (Grant et al., 2014), and has been previously been used as a threshold for high adherence among South African AGYW (Celum et al., 2020). Participants for this specific qualitative subsample were sampled across the entire study, including participants from the control arm (medication pick-up only) and intervention arms (individual or group adherence counselling); participants were also sampled across multiple interview categories (Supplemental Table 2).

The Information-Motivation-Behavioral skills model of behavior change (IMB) (Fisher et al., 2006, 2008) was used to conceptualize and develop questions for the interview guides (Table 1). IMB-informed questions were used to gather information on AGYW-specific phenomena and factors that influence uptake and adherence such as: (a) information, misinformation and concerns, (b) personal and social motivational factors and (c) behavioral skills, tools and strategies needed for both PrEP uptake and adherence. Interviews explored individual PrEP narratives and topics such as enablers and barriers to PrEP adherence, study visit experiences, social support for PrEP use, self-perceived risk over time, and what participants did with unused PrEP. Participants were telephonically approached and invited for an interview. Interviews were conducted in isiXhosa by trained research assistants highly knowledgeable of study community socio-cultural dynamics. Interviews lasted approximately 20–50 min in duration and were completed between September 2019 and April 2021. Interviews were audio-recorded, translated and transcribed in English, and reviewed for quality by qualitative research staff.

**Table 1:** Examples of interview guide questions rooted in the Information-Motivation-Behavioral Skills Model

Model domains	Example interview guide questions
Information	Q. What are ways that you can protect yourself from HIV?
	Q. How would you describe PrEP to a person who has never heard about it before?
	Probe: Who do you think would benefit the most from using PrEP? Why?
	Probe: What doesn’t PrEP do for a person?
Motivation	Q. Can you share with me some of the reasons why you were initially interested in PrEP?
	Probe: What elements helped you to decide to take PrEP?
	Probe: Information, meeting the staff, family, friends?
	Probe: How did you feel about PrEP when you first started?
	Probe: Have your feelings about PrEP changed since you started?
	Q. Have you ever had two or more unopened bottles of PrEP, but continued to come to collect more? Why?
	Probe: What are the reasons you sometimes collect and keep your PrEP, but don’t take it?
Behavioral skills	Q. You have now been on PrEP for _____ months and we want to learn more about your experience while using PrEP
	Probe: Are there any activities that might affect you from taking PrEP every day? What are these?
	Probe: What are ways that you remind yourself to take it? Describe things that would make it easier for you, and other young women, to take PrEP in the future
	Q. You were assigned to the [one-on-one or group adherence counselling arm], can you share with me some of your experiences in your sessions?
	Probe: Can you provide an example of a time when you applied what you learned in the sessions in real life? Why?

### Data analysis

For qualitative analysis, transcripts were coded in Dedoose (2022) following an inductive approach using previously described methods (Mudzingwa et al., 2022a). Codebooks were developed using an iterative approach drawing from review of transcripts and research objectives (Medina-Marino et al., 2021a), considering critical constructs and domains of the IMB model. Code domains covered PrEP knowledge and misconceptions, personal and social motivators for PrEP uptake and adherence, PrEP routines and reminders, PrEP pick-up behaviors and study experiences. The coding team consisted of five researchers [blinded for review] who met weekly to discuss findings, resolve discrepancies, and establish consensus. Given the limited theoretical framing of AGYW PrEP use, we selected a thematic analytical approach. For those with low levels of TFV-DP, code reports containing relevant transcript excerpts were compiled for the following key codes: “Discarding PrEP”, “Sharing PrEP”, “Stockpiling PrEP”, “Selling PrEP”, “Non-PrEP related reasons for study visits”, and “PrEP Recommendations”. Research team members wrote summary memos highlighting themes that emerged from each code report. Given the relatively small sample of interviewed participants with high levels of TFV-DP that met our inclusion criteria, a case approach was followed in which each participant’s transcript was reviewed in depth and summarized in an analytical matrix. The matrix included columns to summarize each High TFV-DP participant’s motivation for using PrEP, social support, PrEP adherence enablers, PrEP adherence barriers, and recommendations for future support interventions. Results from these analyses were discussed by the research team during weekly calls and at a meeting with the larger protocol team (listed co-authors) for critical input.

**Fig. 1:**
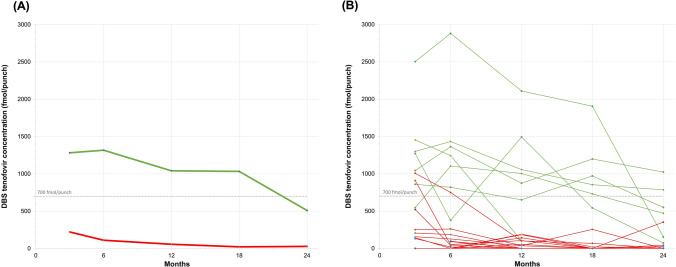
DBS adherence levels for subset of qualitative participants. **A** Average tenofovir-diphosphate levels (TFV-DP) in dried blood spots (DBS) across those with high levels of TFV-DP in their DBS (n = 7; thick green line) and those with low levels of TFV-DP in their DBS (n = 15; thick red line) over the course of the 24-month study. **B** Each line represents the concentration of TFV-DP in DBS for one participant: thin green lines = High TFV-DP participants; thin red lines = Low TFV-DP participants. The grey line in both plots represents the 700 fmol/punch threshold, which corresponds to approximately four oral PrEP doses/week in a USA male population (Color figure online)

Descriptive statistics for the subset of AGYW who completed qualitative interviews was analyzed using Stata 17 (StataCorp, 2021); given the purposive sampling, no additional statistical analysis was conducted. TFV-DP levels in DBS samples at M3, M6, M12, M18, and M24 were graphically displayed to visualize PrEP adherence over time in the study for each qualitative participant included in this subset (Fig. 1; Supplementary Figure S1). The limit of detection for TFV-DP in DBS samples was 16.6 fmol/punch; any measurement below this was counted as zero.

### Ethical considerations

Ethics approval for this research was obtained from the ethics review committee at the University of Cape Town (HREC 289/2018). Separate approval was given by the Eastern Cape Provincial Department of Health to conduct the research in Eastern Cape Province. Written informed consent was obtained prior to participant initiation in the study and prior to each interview. A ministerial waiver of consent was obtained for participants 16–17 years old, with parental consent obtained upon participant request. To ensure confidentiality of study participants, participants’ quotes are represented with TFV-DP level in DBS (High or Low), study site (peri-urban or rural), and age.

## Results

### Participant characteristics and PrEP adherence

Participants’ demographic and behavioral characteristics are summarized in Table 2; these should be interpreted with caution given the small sample size. Those with low levels of TFV-DP were younger (median age 17), were more likely to be attending school and recruited from a mobile pop-up HTS site. A similar percentage from both groups (~ 60%) reported being sexually active during the study. All AGYW with high levels of TFV-DP disclosed their PrEP use to either a family member, partner or friend. Only 67% of those with low levels of TFV-DP disclosed their PrEP use to a family member, partner or friend. Of note, those with high levels of TFV-DP also reported more total PrEP disclosure events and more supportive reactions when they disclosed their PrEP use. Both groups had few participants (n ≤ 2) who self-reported low adherence to PrEP. As measured by TFV-DP in DBS across the two-year study, the average adherence for those with high levels of TFV-DP was over tenfold higher than for those with low levels of TFV-DP (1046 fmol/punch vs. 95 fmol/punch; Fig. 1, Supplementary Figure S1).

**Table 2:** Characteristics of participants in qualitative subset

Characteristic^a^	Low TFV-DP participants	High TFV-DP participants
Number of participants (n)	15	7
Age (median, IQR)	17 (16–22)	22 (19–25)
Site (n, %)		
Urban	7 (46.7%)	3 (42.9%)
Rural	8 (53.3%)	4 (57.1%)
Method of study recruitment (n, %)		
Home visit	2 (13.3%)	5 (71.4%)
Mobile site	13 (86.7%)	2 (28.6%)
Study arm		
Control (PrEP pick-up only)	4 (26.7%)	2 (28.6%)
Adherence counseling (group)	8 (53.3%)	1 (14.3%)
Adherence counseling (one-on-one)	3 (20%)	4 (57.1%)
In school (n, %)	10 (66.7%)	3 (42.9%)
Household size (median, IQR)	4 (4–7)	4 (3–6)
Relation to household		
Head of household	0 (0%)	1 (14.3%)
Family member *e.g. daughter, sister, grandchild*	12 (80.0%)	5 (71.4%)
Adopted / foster / stepchild	1 (6.7%)	0 (0%)
Relative of the household	2 (13.3%)	0 (0%)
Partner / wife	0 (0%)	1 (14.3%)
Taking contraception at baseline	4 (26.7%)	4 (57.1%)
Type of contraception used		
Injectable contraceptives	4 (100.0%)	4 (100.0%)
Ever had sexual intercourse		
At baseline	8 (53.3%)	4 (57.1%)
Between study months 12 – 24^d^	9 (60.0%)	4 (57.1%)
Received incentive^b^ in exchange for sex^c^		
At baseline	4 (26.7%)	2 (28.6%)
Between study months 12 – 24^d^	3 (20%)	3 (42.9%)
Use condoms all or some of the time^c^		
At baseline	8 (53.0%)	3 (42.9%)
Between study months 12 – 24^d^	6 (40.0%)	3 (42.9%)
PrEP disclosure since last interview^d^		
Yes	10 (66.7%)	7 (100%)
No	4 (26.7%)	0 (0.0%)
Refused to answer	1 (6.7%)	0 (0.0%)
Total reported PrEP disclosure events^d^	19	22
People disclosed to about PrEP^d^		
Sex partner(s)	3 (20.0%)	5 (71.4%)
Parent/Guardian	6 (40%)	5 (71.4%)
Sibling	4 (26.7%)	4 (57.1%)
Friend(s)	6 (40%)	4 (57.1%)
Church/ Community leader	0 (0.0%)	2 (28.6%)
Reaction of person disclosed to^d,e^		
Supportive	15 (78.9%)	22 (100.0%)
Not supportive	2 (10.5%)	0 (0.0%)
Neither supportive nor unsupportive	1 (5.3%)	0 (0.0%)
Prefer not to answer	1 (5.3%)	0 (0.0%)
Reported side effects^f^		
Study month 3	2 (15.4%)	0 (0.0%)
Study month 12	3 (27.3%)	2 (28.6%)
Study month 24	3 (20.0%)	0 (0.0%%)
Ever any days that PrEP was not taken (self-reported)^f^		
Study month 3	2 (15.4%)	0 (0.0%)
Study month 12	2 (18.2%)	2 (28.6%)
Study month 24	2 (13.3%)	0 (0.0%)

^a^All responses represent survey data from study baseline unless otherwise specified

^b^Incentives include money, alcohol/drugs, clothes, mobile airtime, place to stay, rides, better marks at school, school fees, food, or anything else

^c^Not applicable amongst participants who reported never having sex or who skipped the question

^d^All of these items were asked at the Month 24 study visit about the past 12 months, except for 1 participant who only had Month 12 behavioral survey data (asking about the past 9 months)

^e^Calculated as percentage across all reported disclosure events for Low TFV-DP v. High TFV-DP participants

^f^Asked whether experienced in the time period preceding the study visit (e.g., either in the past 3, 9, or 12 months for Month 3, Month 12, and Month 24 study visits, respectively). Missing data: Month 3 (n = 2 Low TFV-DP participants); Month 12 (n = 5 Low TFV-DP participants); Month 24 (n = 1 High TFV-DP participant)

### Low TFV-DP participants: mistrust and shame as primary barriers to PrEP use

AGYW with low levels of TFV-DP highlighted barriers to PrEP use including negative talk casting doubt on PrEP, misconceptions, and mistrust of PrEP by members of their social networks and community. For example, one woman said that she considered discarding PrEP because of “*the way people were talking about it – it was like a disease pill*” (Low, peri-urban, age 16). Another described reasons why others were not using PrEP: “*not trusting this PrEP if it’s working or maybe being told bad things about PrEP by the friends but not wanting to come and cancel herself here at the study”* (Low, peri-urban, age 23)*.* Other Low TFV-DP participants described a lack of support from family or partners, feeling judged by others, unresolved side effects which were not discussed with study staff, and an overall lack of interest in PrEP. One described how her brother (her “*support system*” in life) accused her of being HIV positive because she was using PrEP: “*when I started using PrEP he [brother] use to swear at me saying that I’m using ARVs and I’m hiding them*” (Low, rural, age 16). Low TFV-DP participants described how their social networks were unfamiliar with PrEP, questioning its purpose and effects on the body.

### Non-PrEP-related reasons for study visits: material benefits, service delivery, social desirability

Many individuals with low levels of TFV-DP openly shared their own and others’ motivations for attending study visits despite not using PrEP, citing other benefits including reimbursement money, snacks, sanitary pads, and HIV and pregnancy tests. As one participant shared, *“Some of them you would hear that they won’t actually talk about the pill, they would say I need that money or I have slept with that guy so I don’t know that I am not pregnant or HIV positive, so some other reasons they come for something else, they want to benefit”* (Low, peri-urban, age 22).

Low TFV-DP participants from the rural study site described more examples of being in “*desperate*” situations, with greater lack of resources than those from the peri-urban site, as influencing their study visit attendance. Rural participants shared how some AGYW came *“when they are desperate of money that’s when they decide to come and take PrEP”* (Low, rural, age 16*); “they are desperate for that R30 [$2 USD]”* (Low, rural, age 23). Another said, *“maybe there is nothing to eat at home … maybe will start thinking that I can come to PrEP to collect R30 [$2 USD]… or maybe she is craving for chips”* (Low, rural, age 16). Peri-urban participants were also motivated to come due to money, snacks, and sanitary pads, but those participants did not elaborate on what difference that might make in their lives the way some rural participants did.

AGYW described receiving better health services at the study site (e.g., friendlier staff, shorter queues) compared with public clinics as a significant motivator. Others shared how the group or one-on-one PrEP adherence sessions motivated them to come for PrEP study visits. Notably, many shared that the most valuable part of the PrEP adherence sessions was not the PrEP-specific education they received, but rather the opportunity to learn career and goal-setting skills (e.g., interviewing, CVs) and to discuss interpersonal problems they were facing at home.

Some AGYW also described peer pressure from friends to visit the site, and social desirability of not wanting to disappoint study staff by missing study visits. For example, one participant described not feeling at risk for HIV, but still wanting to come to the study: “*Because I did not have a boyfriend, so I did not see the reason to take it. But I was continuing coming* […] *I didn’t want to skip my date here. I wanted to continue with my study nicely”* (Low, peri-urban, age 23).

### Unpacking “unique patterns” of PrEP use: driven by anticipated stigma, perceived PrEP need for others, and seasons of risk

AGYW with low levels of TFV-DP described what happened to unused PrEP, including stockpiling, discarding, selling, and sharing PrEP (see Table 3 for representative quotations for each pattern).

**Table 3:** Illustrative quotations from Low TFV-DP participants describing what they did with their PrEP

**Stockpiling PrEP**
“*I think they were 6 [unopened PrEP bottles at home] then, now I am left with 3. […] Cause maybe I am not going to make after 5 years … like I am also growing like my time is running out I will also get to 25 [maximum age for study eligibility.]”* (Low, peri-urban, age 16) *“Maybe she has side effects and she don’t care, as people we not the same. There are people that can’t tell the nurses that they have side effects when they take [PrEP]. So they think they should go and collect it and stockpile it.”* (Low, rural, age 16) “*Maybe a person want this R30 [$2 USD] [reimbursement] or they lack support from home […] They do continue to collect the pills and just keep them or their boyfriend don’t want them to take PrEP.”* (Low, rural, age 23)
**Discarding PrEP**
*“I flush [PrEP pills down the toilet]. […] [I] am avoiding maybe people who like dumps, dogs. Or others will think [I] am taking ARVs even though [I] am not taking them. They will not understand maybe when I throw it in the bin maybe dogs can come and spill and tear the dump bin.”* (Low, peri-urban, age 21) *“I have witnessed that, I was walking with this person I don’t want to mention their name and she threw it away at the road. She opened it and threw it away. I asked her why, and she said she has side effects and I said to her when you feel that way you should tell the nurses [you] feel sick when you taking PrEP. And she said she won’t do that.”* (Low, rural, age 16) “*People are embarrassed to take them and they don’t take the pills. […] Maybe it is because people are unfamiliar with the pills, it was also my first time seeing them. And as we keep getting informed about them, some don’t come and end up not getting information on what PrEP is exactly. So the person end up messing up and saying ‘no you are lying, these pills are for something else.’”* (Low, peri-urban, age 16) *“I asked myself that why do I take this [PrEP] but I don’t have a boyfriend?”* Said she threw pills *“in the dust bin because I had no boyfriend at that time.”* (Low, peri-urban, age 22)
**Sharing PrEP**
*“I have shared it with someone. It was the whole box, I did not use it, and it was my cousin who was living at the rural areas. While I was telling her about it she was like ‘I want this but where I stay there is no one that teaches us about these things’. I had to that share that box. In fact she does not have one partner and she is not using condoms, so I was like you know what let me to introduce you to this so that every time you come here I will take you to the container.”* (Low, peri-urban, age 22) “*When my PrEP is finished I take from my sister before I come and collect mine here. Let’s say I’m supposed to come the site on the 15th and I forget my date on the 15th and I don’t come, then my PrEP gets finished, I take from S. [her sister] then the following day I go to the site to collect my pills. That’s how we share.”* (Low, rural, age 24) *“The ones that are curious… maybe those are the ones that like to come [for PrEP]. And then there are those friends of theirs then that are not curious […] because they are shy for going there to that PrEP container. Because they know everyone here in the community that PrEP container what it does. […] doesn’t want to be seen by her boyfriend… maybe her boyfriend has criticized the container.”* (Low, peri-urban, age 22)
**Selling PrEP**
*“So then I don’t want to tell on others but there’s one of our participants that has been selling PrEP, selling to this one guy. […] There was a guy that came to me and he asked me how can he get these pills and so forth, and I said to him it’s only young girls that can get them and he said there is a participant that comes and collects her PrEP, and he’s staying this side, so he asked me about her.”* (Low, rural, age 24) *“They could sell PrEP at a faraway places maybe […] they want to make money out of them, they think if they have this much they will charge R120 or R140 [$8–9 USD] when they are three [bottles of PrEP].”* (Low, peri-urban, age 16)

^*^Participant quotations are labeled with adherence level based on DBS, study site, and age at baseline

#### Stockpiling PrEP

Stockpiling PrEP was defined as times when participants kept multiple unopened or partially used bottles of PrEP at home. Several participants described personally stockpiling PrEP due to missing doses or taking a break from PrEP. During these times, participants decided to keep PrEP instead of discarding it. One participant mentioned concerns about not having access to PrEP in the future when she might really need it, and another alluded to event-driven use of PrEP when traveling to visit a boyfriend who stays far away. When probed by the interviewer, most participants agreed that others stockpile because they are (1) not currently in a relationship (low perceived risk), (2) are not interested in PrEP, or (3) are taking a break from PrEP. A few participants thought other AGYW might be saving PrEP for future seasons when they might be at higher risk or were afraid they would not have access to PrEP in the future due to clinic barriers.

#### Discarding PrEP

Discarding PrEP was consistently described by Low TFV-DP participants as a response to not actually wanting PrEP but attending site visits for other benefits. Two participants disclosed personally discarding PrEP themselves, while the majority of those with low levels of TFV-DP described having conversations with or personally witnessing other participants throwing away PrEP. When probed about hypothetically where they would throw PrEP away, participants suggested trash bins, in the street or toilet, or locations near the study site. Their descriptions point to stigma around PrEP use and concern about other people seeing evidence of them taking pills. For some, this meant discarding PrEP near the study site where no one in the community would see them with pills, or in the toilet where there was more privacy from family members. For example, one participant said she would throw PrEP in the toilet due to concern about her mother finding it in the trash bin. Others highlighted concern for children or animals finding the pills and eating them accidentally.

#### Sharing PrEP

Participants described PrEP being shared with sisters, cousins, or boyfriends who were perceived to be at higher risk for PrEP than themselves, or who did not meet eligibility criteria to join the study. Three participants gave examples of personally sharing PrEP – one with a cousin in the rural areas who was perceived to be at higher risk for becoming infected with HIV, and another with her sister who was also a CPS study participant. Some described sharing PrEP with friends who wanted PrEP but who were too embarrassed to come to the site themselves or lived far away. This suggests that a number of barriers (e.g., stigma; distance from clinics / study sites) interfere with accessing or using PrEP. However, descriptions of sharing PrEP with friends indicate that some in the study communities saw value in using PrEP.

#### Selling PrEP

There were relatively few instances of participants selling PrEP, but two participants gave descriptive answers when probed about if others might be selling PrEP. One participant thought that others may sell PrEP at faraway places, giving a specific price range for selling PrEP. Another participant said she knew of a fellow participant who had been selling PrEP to a man outside the study, but lacked specific details when probed. This participant also commented that she did not want to be “*telling on anyone*”, signifying potential shame associated with selling PrEP and fear of disapproval from research staff.

### High TFV-DP participants: social support, trust in PrEP, and planning skills enabled high PrEP adherence over two years

In contrast to those with low levels of TFV-DP in their DBS, participants who had high levels of TFV-DP described what motivated them to adhere to PrEP over a two-year time period (see Table 4 for representative quotations).

**Table 4:** Illustrative quotations from High TFV-DP participants describing factors enabling their PrEP use

**Supportive partners and family members**
*“Even my boyfriend I told that I’m taking PrEP, he’s my supporter. He even said when I go to the gym ‘take your pill box put them in your bag you will drink it at the gym’, and when the alarm rings at 8 pm and we are at the gym. […] Even my grandmother said “no, if you see that it’s [PrEP] good for you, take it my child.”* (High, peri-urban, age 23) *”Yes at home they [family] know about it because when I started using PrEP they [study staff] came to my home and I was with them.* […] *At home I have a sister who is [living with] HIV, so my mother said, ‘no I can see it [PrEP] is a right thing and it can also help you’ and I also had that in my mind. […] It's not something that I hide … it’s something that will help me.”* (High, rural, age 25) *“I told my sister, and she was supportive. She wanted it for my other sister’s daughter but they had already stopped taking new people. She also wanted it for herself […] I wanted them to know and also advise me if I did something that is out of line.”* (High, peri-urban, age 22) *“They [family] asked a lot of questions to the person who was introducing PrEP to me [study staff member during home visit]. The counsellor told my mother that PrEP is safe to use and a lot of people are using it, it was not the first time she heard about PrEP. […] it was not difficult to convince her."* (High, rural, age 25)
**Belief that PrEP works**
*“My friend says I should not take PrEP, it’s wrong for me, your boyfriend will leave you, those pills will give AIDS. […] I told her that she will regret when she has AIDS that why didn’t she take PrEP, and as for me I will die without contracting AIDS.”* (High, peri-urban, age 23) *"I used to [feel at risk] before PrEP but now I feel free because PrEP is my hope, I take it every day, I never miss it. […] I will always use it because my partner has HIV and I would like to continue with it."* (High, peri-urban, age 22) *“When PrEP was introduced there were people who said bad things about it. [….]* ‘*These pills are going to cause disease’ or ‘they are not accurate’ or ‘they have side effects’.* […] *They said so many things but on my mind I thought they are not the ones who introduced me to PrEP […] They don’t know anything about it. They are hearing it by those who have not learned about it. So I thought it is better to listen to these people [study staff] than to listen to people who do not know about it. […] I will see it by myself … and if they [PrEP pills] have side effects I will tell PrEP study staff and they will tell me what to do.”* (High, rural, age 25) *“[PrEP] was protecting me from getting HIV virus so that is one thing that drove me."* (High, peri-urban, age 18)
**Planning for PrEP taking**
*“[When first started PrEP] I usually forgot about it [PrEP], and I thought to myself that it will help me to set an alarm and I should not hide it. I put it on top of the dresser in the morning […] then I see it.”* (High, rural, age 25) *"I keep [PrEP] in the wardrobe because that is the only place I open it everyday. […] When I used to forget I set an alarm. These days on your smart phone you can write what is that alarm for, I used to write “take PrEP” alarm. […] We have that brown container [pill box] so even if I am not at home I still take my pills with, there is not anything that prevents me from taking PrEP.”* (High, peri-urban, age 18) *“I keep it on that green container I got from here, next to the children’s lunch boxes in the morning at home. So that I can always see it and not forget it. […] I have an alarm that rings at 8 pm, so when that alarm rings I know it’s time to take PrEP, and when my phone is off I have a ring that I wear and that ring I put it on my pinkie finger. When I put it on my pinkie finger it reminds me to always ask what time it is.*" (High, peri-urban, age 23)
*Participant quotations are labeled with adherence level based on DBS, study site, and age at baseline

#### Role of supportive partners and family members

High TFV-DP participants were initially motivated to use and persist with PrEP due to relational support from partners and/or household members. For example, one High TFV-DP participant has been living with her HIV positive partner (and father of their child) for four years. She described how he contributed to her decision to start PrEP and supported her throughout the study: *“I was interested because there was someone who was supportive […] He is able to come along to my PrEP dates [study visits]. He reminds me about my dates and asks me how they treating me at the container [study site]”* (High, peri-urban, age 22). All four individuals with high levels of TFV-DP from the rural site described how their families were present when PrEP was being introduced during the home-based HTS visit, and encouraged them to start and continue using PrEP. Other High TFV-DP participants discussed their motivation to protect themselves from HIV because they grew up in households with someone living with HIV. Notably, all High TFV-DP participants disclosed PrEP use to people they were close with, including partners and family members. These AGYW described how they did not need to hide anything, including their PrEP use, from household members – *“They’ve always known since the first day I started*” (High, rural, age 21) or “*I signed everything in front of them; they know it and also my friend knows it"* (High, rural, age 25). High TFV-DP participants also described how family members and partners helped remind them to take PrEP and attend their study visits.

#### Belief that PrEP works

Most AGYW with high levels of TFV-DP were first introduced to PrEP by study staff during HTS activities, and then continued to learn more throughout study visits. They described study staff as reliable and trustworthy sources of knowledge (more than their peers), and as "friends" or "big sisters" with whom they felt comfortable talking openly with throughout the study. These AGYW further described trusting their own knowledge of HIV and PrEP. This was in contrast to the PrEP misconceptions and lack of knowledge they attributed to their peers and communities. They further described encountering misconceptions from friends about PrEP, but remained confident in their own knowledge. Several assertively pushed back against misconceptions and advocated for PrEP. For example, one young woman with high levels of TFV-DP described how she would convince others about PrEP: “*It [PrEP] works. […] I believe if you sleep with someone who is HIV positive it will be effective. […] If you take it daily it will be effective but if you skip a week it will not be effective because it is decreasing in your blood so you have to take it daily so that it will make the shield stronger"* (High, peri-urban, age 18). This young woman specifically mentioned wanting to continue taking PrEP after the study ends because she believes it works to protect her from HIV.

#### Planning for PrEP taking

AGYW with high levels of TFV-DP described having strict, established routines for taking PrEP, being supported with reminders, and/or planning ahead for how to take PrEP when away from home. Many described their PrEP taking routine in great detail: what time they would take PrEP every day, storing PrEP in a place in the open where they would regularly see it, and using alarms to remind them to take it. These routines seemed to have developed and strengthened over time. Some of these AGYW described overcoming an initial period of forgetting to take their PrEP by having family members or partners remind them. For example, one participant reflected on how she became a more consistent PrEP user over the study: *“I was very lazy but I knew that I don’t want to have HIV. As months went then I got used to it, I took it every day and I enjoyed it, yeah at first I would really forget but got used to it”*(High, peri-urban, age 18). Some High TFV-DP participants described strategizing for how to remember PrEP when away from home, such as this young woman: *"If I know that I’m going away I know that my pill box must be in my pocket so that I can take my PrEP when the time comes"* (High, peri-urban, age 23).

### Need for increased awareness campaigns and familial support to reduce shame and misconceptions: recommendations from participants to improve future PrEP adherence

Both those with low and high levels of TFV-DP in their DBS shared multiple suggestions for how to improve PrEP uptake and adherence in their communities. Among AGYW with low TFV-DP, many recommended increasing education in schools and clinics to counteract PrEP misconceptions: “*Go to schools and tell them more about PrEP and what kind of a pill it is. Because most people believe that PrEP is something else. Or when you go to the clinic there could be people there to explain to people about PrEP and how to take PrEP”* (Low, rural, age 16). Some Low TFV-DP participants thought longer-acting PrEP methods like an injection or once-monthly pill would improve use of PrEP, such as this woman: “*If they can be injected with PrEP they can come. They can’t say I took it while they did not when they are injected”* (Low, rural, age 16).

Those with high levels of TFV-DP recommended improving relational support from family members and friends for PrEP use and broad campaigns to improve PrEP knowledge in communities. One young women with high levels of TFV-DP suggested that for someone to succeed in using PrEP, “*a person must consult her parents so that her parents can understand and also her friend so that they may do the same thing*” (High, rural, age 25). To increase knowledge about PrEP, other High TFV-DP participants recommended PrEP awareness campaigns to clearly explain what PrEP is, how it works, and who it is for. For example, one participant suggested using posters, community committees, and participants “*that are accurate on PrEP*” who can explain the benefits of PrEP to others and counteract “*some people misinterpret PrEP*” (High, peri-urban, age 23). While High TFV-DP participants said they preferred picking up PrEP at the study site due to friendly staff and quick pick-up, most said they would still pick up PrEP at the public clinic in the future.

Another High TFV-DP participant recommended that her peers need to overcome feelings of shame associated with taking PrEP: “*A person must not have pride and do what is right for herself. [,,,] They should not be ashamed; they should put pride aside. A person doesn’t know what tomorrow brings. This is the way a person should be safe*" (High, rural, age 25). For her personally, the one-on-one counseling sessions at the study site helped to raise her own awareness of peer pressure and “*focus on what I do and use my time for the right thing*s” (High, rural, age 25).

## Discussion

This qualitative sub-study evaluated PrEP use experiences of 22 South African AGYW who had high return rates for monthly PrEP refills over two years in the Community PrEP Study, but drastically different levels of TFV-DP in their DBSs. Those with low levels of TFV-DP were primarily influenced by misinformation, negative PrEP perceptions from peers and/or family members, and low self-perception of HIV risk. In response to these barriers, those with low levels of TFV-DP described how they would discard PrEP to avoid stigma, stockpile PrEP for future seasons of risk, or share PrEP with others who had higher perceived risk for HIV. These same AGYW were motivated to continue participating in the study because of other study benefits such as better quality health care, reimbursement money, and not wanting to disappoint peers or study staff. In contrast, those with high levels of TFV-DP described a strong belief that PrEP works to protect them from HIV, high levels of social support from their families and/or partners, and behavioral skills which allowed them to plan ahead for persistent PrEP use. All participants recommended improving future PrEP adherence through community PrEP awareness campaigns, use of peer advocacy akin to a “PrEP Champion” (Amico et al., 2017; Haberer et al., 2019), and better engagement with familial and social networks.

Other study benefits were a powerful motivator for AGYW to participate in this PrEP study, even if they had no interest in personally using PrEP. Previous PrEP clinical trials in South Africa, including VOICE and FEM-PREP, also found that women with low drug adherence levels were motivated by reimbursements and ancillary health care to continue study participation (Corneli et al., 2015; Stadler et al., 2016). In our study, we observed that women from our rural community site described in more detail how provisioning of reimbursement money, snacks, and sanitary pads provided by the study motivated their participation. This may point to a difference in drivers for study participation between peri-urban and rural settings, as AGYW in rural settings have less economic opportunities for even small amounts of money compared to their peri-urban counterparts. Consistent with VOICE trial findings, our study highlights HIV risk factors, such as poverty and social inequalities, may also motivate participation in clinical studies (Stadler et al., 2016). It also raises the importance of identifying felt needs of AGYW in study communities (e.g., food, menstrual health products, social support and mentorship) and integrating these needs into multi-level interventions including HIV prevention in the future. Future studies that do not make participation contingent on HIV prevention use may allow for more authentic engagement. More research is needed on how to make HIV prevention a priority among those at highest risk, such as framing PrEP use as sex and/or health positive and increasing peer and family support for PrEP.

In our study, discarding/throwing away PrEP was the most frequently described pattern of managing unused PrEP by those with low levels of TFV-DP. These young women also highlighted the role of negative talk about PrEP among other participants and their social networks, thus leading to mistrust of PrEP. Other qualitative studies on PrEP use among women in South Africa reported similar findings, including participants being demotivated by negative “waiting room talk” (Straten et al., 2014) and high levels of community misunderstanding of PrEP (Rousseau et al., 2021; Straten et al., 2014). Discussion by study participants regarding where PrEP was discarded (e.g. trash bin, street, toilet) highlighted stigma around PrEP use. Furthermore, similar to VOICE trial participants, young women in our study expressed concern about other’s perceptions regarding pill taking (Stadler et al., 2016; Straten et al., 2014). We observed that community-level misinformation and lack of awareness about PrEP affected adherence. This was likely due to PrEP still being new and relatively unknown in the Eastern Cape province study communities. Our results and participant voices point to the need for new strategies to quickly build trust and confidence in new medical interventions, such as engagement with schools and peer-led PrEP awareness campaigns. This is likely doubly important with the expected approval and roll out of long-acting injectable cabotegravir.

All our study participants with high levels of TFV-DP described disclosing their PrEP use to supportive family and/or partners. This is consistent with other PrEP studies in South Africa which reported that women who disclosed to supportive social networks had better adherence (Giovenco et al., 2021; O’Rourke et al., 2021; Stoner et al., 2021; Straten et al., 2014). We have previously reported on the role of engaging families during home visits prior to PrEP initiation as a helpful component for building family support (Medina-Marino et al., 2021a) and the importance of disclosure for improving PrEP adherence (Daniels et al., 2021c). Our present analysis further confirms the need for engagement with family members and social support networks from an early stage in PrEP interventions for AGYW. It also highlights the need for PrEP interventions that specifically support younger AGYW ages 16–19 to persist with PrEP. For those with low levels of TFV-DP, high levels of study support (e.g., monthly visits, adherence counselling, and supportive study staff) did not translate to high levels of PrEP use for those in this sub-study. Additional measures are needed in PrEP interventions for younger AGYW including social support at home and lessened stigma around PrEP use among peers and in the community. Our research also highlights the need for helping AGYW access menstrual hygiene products and other health services if they do not want PrEP.

In contrast to those with low levels of TFV-DP, study participants with high levels of TFV-DP described having a high level of trust that PrEP worked to protect them. Trust in PrEP and those providing it was a key factor among women who accepted PrEP in the HPTN067/ADAPT qualitative sub-study among young women in Cape Town, South Africa (Amico et al., 2017). As presented in the Mutuality Framework in HPTN067/ADAPT, those in the Alignment and Mutuality Dynamics demonstrate strong beliefs that PrEP is effective and are able to overtly challenge PrEP misconceptions expressed by others (Amico et al., 2017). We observed similar qualities among High TFV-DP participants in this study, as they assertively countered misinformation from peers. Future PrEP persistence interventions should consider how to promote trust in PrEP, habit formation, and long-term planning skills among AGYW.

This study had several limitations. First, our study included a relatively small sample size, especially among those with high levels of TFV-DP. However, most of the interviews conducted with AGYW with high levels of TFV-DP were highly descriptive and conducted later in the study, allowing participants to reflect back across 18 + months of study experiences. Second, since interviews were conducted by site study staff, social desirability bias may have limited openness of responses about personal reasons for study participation and low PrEP adherence. To reduce this bias, interview questions were framed so that participants could share their peers’ experiences instead of personal experiences.

## Conclusions

This study is one of the first to compare PrEP adherence among South African AGYW over a longer time frame (two years) and report on nuances of unique behaviors relating to the handling of PrEP, including stockpiling, sharing, and selling PrEP. Our results have several implications for future PrEP intervention design among AGYW in South Africa and similar settings. First, our results indicate the need for social support interventions which increase PrEP awareness among families and household members, particularly among younger AGYW ages 16–19. Engaging with familial and social networks of AGYW starting at the point of PrEP introduction may facilitate and action support for more optimal prevention-effective adherence. Second, this research highlights the need for ethical considerations regarding incentives to reduce participation solely for other study benefits while still appropriately compensating participation. Finally, interventions are needed to minimize stigma associated with visiting a research site or clinic and taking daily pills. For example, these may include alternate delivery mechanisms (e.g., non-clinic-based PrEP collection), long-acting PrEP forms which allow for discreet use, and multi-faceted community education developed together with AGYW.

### Supplementary Information

Below is the link to the electronic supplementary material.

Supplementary file1 (doxc 106 kb)

Supplementary file2 (doxc 27 kb)

Supplementary file3 (doxc13 kb)

## Data Availability

De-identified data presented in this manuscript will be shared upon reasonable request and receipt of a completed data request form.
