# Supplementary material for: High study participation but diverging adherence levels: qualitatively unpacking PrEP use among adolescent girls and young women over two years in Eastern Cape, South Africa
Source: J Behav Med. 2023 Dec 11;47(2):320–33. doi: 10.1007/s10865-023-00462-2 (PMC10944421; doi:10.1007/s10865-023-00462-2)
Supplement: Supplementary file 1 — Supplementary file1 (doxc 106 kb) [file 10865_2023_462_MOESM1_ESM.docx]

**Supplementary Figure S1. Qualitative subset adherence levels over 24 months, as measured by tenofovir-diphosphate (TFV-DP) in dried blood spots (DBS).**
