# Supplementary material for: High study participation but diverging adherence levels: qualitatively unpacking PrEP use among adolescent girls and young women over two years in Eastern Cape, South Africa
Source: J Behav Med. 2023 Dec 11;47(2):320–33. doi: 10.1007/s10865-023-00462-2 (PMC10944421; doi:10.1007/s10865-023-00462-2)
Supplement: Supplementary file 2 — Supplementary file2 (doxc 27 kb) [file 10865_2023_462_MOESM2_ESM.docx]

**Supplemental Table 1. Consolidated criteria for reporting qualitative studies (COREQ): 32-item checklist for interviews and focus groups**

Developed from: Tong A, Sainsbury P, Craig J. Consolidated criteria for reporting qualitative research (COREQ): a 32-item checklist for interviews and focus groups. International Journal for Quality in Health Care. 2007. Volume 19, Number 6: pp. 349 – 357

| Item No. | Topic | Guide Questions/Description | Reported on  Page no. |
| --- | --- | --- | --- |
| Domain 1: Research team and reflexivity | | | |
| *Personal Characteristics* | | | |
|  | Interviewer/  facilitator | Which author/s conducted the interview or focus group? | **Methods, pg. 5**  “Interviews were conducted in isiXhosa by trained research assistants…” under the supervision and support of authors MM, LF and LDV |
|  | Credentials | What were the researcher’s credentials? E.g., PhD, MD | LGB: MBChB, DTMH, DCH, FCP  AMM: PhD, MSc |
|  | Occupation | What was their occupation at the time of the study? | Principal investigators |
|  | Gender | Was the researcher male or female? | Female and male |
|  | Experience and training | What experience or training did the researcher have? | LGB: Physician scientist and infectious disease specialist. Research focus areas include programmatic and action research around ARV roll out and TB integration, prevention of HIV in women, youth and men who have sex with men.  AMM: Molecular biologist and public health researcher with significant training and experience in field epidemiology, program evaluation, implementation science and systems strengthening activities |
| *Relationship with participants* | | | |
|  | Relationship established | Was a relationship established prior to study commencement? | Researchers had no relationships with participants. Research assistants were known by participants through study follow-up calls and administrative issues but did not have the same established relationship as other staff. |
|  | Participant knowledge of the interviewer | What did the participants know about the researcher? E.g., Personal goals, reasons for doing the research | **Methods, pg. 6**  “Written informed consent was obtained prior to participant initiation in the study and prior to each interview.”  The reason for the research was introduced by research assistants and explained using an informed consent form. |
|  | Interviewer characteristics | What characteristics were reported about the interviewer/ facilitator? E.g., Bias, assumptions, reasons, and interests in the research topic | **Methods, pg 5.**  “Interviews were conducted in isiXhosa by a trained research assistant highly knowledgeable of study community socio-cultural dynamics” |
| Domain 2: Study Design | | | |
| Theoretical framework | | | |
|  | Methodological orientation and Theory | What methodological orientation was stated to underpin the study? e.g., grounded theory, discourse analysis, ethnography, phenomenology, content analysis | **Methods, pg. 5**  **“…**The Information-Motivation-Behavioral skills model of behavior change [29,30] was used to conceptualize and develop questions for the interview guides…”  **Methods, pg. 5**  “…Given the limited theoretical framing of AGYW PrEP use, we selected a thematic analytical approach.” |
| Participant selection | | | |
|  | Sampling | How were participants selected? e.g., purposive, convenience, consecutive, snowball | **Methods, pg. 4**  “A total of 89 participants were purposively recruited to complete qualitative interviews to further explore their experiences initiating and adhering to PrEP throughout the study” |
|  | Method of approach | How were participants approached? e.g., face-to-face, telephone, mail, email | **Methods, pg. 4**  “CPS participants were telephonically approached and invited for an interview.” |
|  | Sample size | How many participants were in the study? | **Methods, pg. 4**  “…This manuscript examines a subset of (n=22) these participants who all had high rates of return for monthly PrEP refills, and attended at least three of five study visits in which DBS samples were collected.” |
|  | Non-participation | How many people refused to participate or dropped out? Reasons? | N/A, some eligible participants could not be successfully contacted for an IDI or had relocated / some participants that were invited telephonically did not present for an IDI. |
| Setting | | | |
|  | Setting of data collection | Where was the data collected? E.g., home, clinic, workplace | In a private space at the study site |
|  | Presence of nonparticipants | Was anyone else present besides the participants and researchers? | IDIs were conducted one-on-one with the interviewer |
|  | Description of sample | What are the important characteristics of the sample? e.g., demographic data, date | **Methods, pg. 4**  “…We then stratified these 22 study participants into two groups: 1) those (n=7) with high levels of TDF-DP in DBS (≥700 fmol/punch) averaged across all their DBS measurements during the study (“High TFV-DP”), and 2) those (n=15) with low to moderate levels of TDF-DP in DBS (<700 fmol/punch) averaged across all their DBS measurements during the study (“Low TFV-DP”).”  **Methods, pg. 5**  “Interviews were completed between September 2019 and April 2021.”  **Results, pg. 7**  “Participants’ demographic and behavioral characteristics are summarized in Table II.“ |
| Data collection | | | |
|  | Interview guide | Were questions, prompts, guides provided by the authors? Was it pilot tested? | **Methods, pg. 5**  “The Information-Motivation-Behavioral skills model of behavior change [29,30] was used to conceptualize and develop questions for the interview guides (Table I). IMB-informed questions were used to gather information on AGYW-specific phenomena and mentioned factors that influence uptake and adherence such as issues related to: a) information, misinformation and concerns, b) personal motivational and social motivational factors and c) behavioural skills, tools and strategies needed for both PrEP uptake and adherence.”  All interview guides were translated into isiXhosa and checked for accuracy. |
|  | Repeat interviews | Were repeat interviews carried out? If yes, how many? | **Supplemental Table 2 (see footnote).**  For this sample n=1 with repeat interviews was included (interviewed as part of multiple interview categories). |
|  | Audio/visual recording | Did the research use audio or visual recording to collect the data? | **Methods, pg. 5**  “Interviews were audio-recorded, translated and transcribed in English, and reviewed for quality by qualitative research staff.” |
|  | Field notes | Were field notes made during and/or after the interview or focus group? | N/A |
|  | Duration | What was the duration of the interviews or focus group? | **Methods, pg. 5**  “Interviews lasted approximately 20-50 minutes in duration” |
|  | Data saturation | Was data saturation discussed? | **Methods, pg. 4-5.**  For this qualitative sample, there were 22 participants that we qualitatively interviewed who met our definition for high rates of return for monthly PrEP refills with at least three DBS measurements; we included all 22 in this analysis. These participants were then stratified according to TFV-DP levels.  **Methods, pg. 6**  “Results from these analyses were discussed by the research team during weekly calls and at a meeting with the larger protocol team (listed co-authors) for critical input.” |
|  | Transcripts returned | Were transcripts returned to participants for comment and/or correction? | Transcripts were not returned to participants. However, transcripts were reviewed for quality by qualitative research staff. |
| Domain 3: analysis and findings | | | |
| Data analysis | | | |
|  | Number of data coders | How many data coders coded the data? | **Methods, pg 5.**  “The coding team consisted of five researchers (MA, JD, LDV, LF, EKM) who met weekly to discuss findings, resolve discrepancies, and establish consensus.” |
|  | Description of the coding tree | Did authors provide a description of the coding tree? | **Methods, pg. 5**  “For qualitative analysis, transcripts were coded in Dedoose [31] following an inductive approach using previously described methods [21]. Codebooks were developed using an iterative approach drawing from review of transcripts and research objectives [19], considering critical constructs and domains of the IMB model.” |
|  | Derivation of themes | Were themes identified in advance or derived from the data? | **Methods pg. 6**  “Research team members wrote summary memos highlighting themes that emerged from each code report. Given the relatively small sample of interviewed participants with high levels of TFV-DP that met our inclusion criteria, a case approach was followed in which each participant’s transcript was reviewed in depth and summarized in an analytical matrix.” |
|  | Software | What software, if applicable, was used to manage the data? | **Methods, pg. 5**  “For qualitative analysis, transcripts were coded in Dedoose [31] …” |
|  | Participant checking | Did participants provide feedback on the findings? | N/A |
| Reporting | | | |
|  | Quotations presented | Were participant quotations presented to illustrate the themes / findings? Was each quotation identified? E.g. Participant number | **Methods, pg. 6**  **“…**participants’ quotes are represented with TFV-DP level in DBS (High or Low), study site (peri-urban or rural), and age.” |
|  | Data and findings consistent | Was there consistency between the data presented and the findings? | **Methods, pg. 6**  “Results from these analyses were discussed by the research team during weekly calls and at a meeting with the larger protocol team (listed co-authors) for critical input.” |
|  | Clarity of major themes | Were major themes clearly presented in the findings? | **Results, pg. 7-13**  Findings and participant quotes have been presented under major themes/headings in the results |
|  | Clarity of minor themes | Is there a description of diverse cases or discussion of minor themes? | **Results, pg. 8, 10-11**  Yes, minor themes include low TFV-DP participants from the rural site describing how lack of resources influenced their study visits compared with high TFV-DP participants. Other minor themes include some unique patterns of PrEP use such as selling PrEP, as described in results. For example:  “There were relatively few instances of participants selling PrEP, but two participants gave descriptive answers when probed about if others might be selling PrEP.” |
