# Supplementary material for: High study participation but diverging adherence levels: qualitatively unpacking PrEP use among adolescent girls and young women over two years in Eastern Cape, South Africa
Source: J Behav Med. 2023 Dec 11;47(2):320–33. doi: 10.1007/s10865-023-00462-2 (PMC10944421; doi:10.1007/s10865-023-00462-2)
Supplement: Supplementary file 3 — Supplementary file3 (doxc13 kb) [file 10865_2023_462_MOESM3_ESM.docx]

**Supplemental Table 2. Interview categories represented in this qualitative analysis**

| **Interview category** | **Definition** | **Number of interviews included in this qualitative sub-analysis*** |
| --- | --- | --- |
| Unique patterns | Participants identified by study staff as having a unique pattern of medication use, including high study participation, but low drug adherence | 13 |
| Study arms | Interviews conducted at Months 15-24 about experiences in one of three study arms (control – medication pick-up only, one-on-one adherence counseling, or group adherence counseling) | 6 |
| Serial interviews | Participants who were interviewed at Months 1-3 and again at Months 18-24 about their study experiences | 1 |
| Immediate presenters | Participants who presented to study sites for PrEP initiation within 0-3 days of completing baseline questionnaire | 3 |

*One participant was interviewed twice, once in the “Study Arms” category and once as the “Serial Interviews” category
